# Adjuvant therapy for operable breast cancer; more answers, new questions.

**DOI:** 10.1038/bjc.1995.223

**Published:** 1995-06

**Authors:** T. J. Perren


British Journal of Cancer (1995) 71, 1142-1144

© 1995 Stockton Press All rights reserved 0007-0920/95 $12.00

EDITORIAL

### Adjuvant therapy for operable breast cancer; more answers, new questions

TJ Perren

*ICRF Cancer Medicine Research Unit, St. James's University Hospital, Leeds, UK. On behalf of the ABC Trial Steering Committee**

The first overview of systemic adjuvant treatment for early breast cancer by The Early Breast Cancer Triallists' Collaborative Group (EBCTCG) was published in 1990. It demonstrated, over a 5 year period, a significant improvement in mortality for women over the age of 50 when treated with tamoxifen and for women under the age of 50 when treated with chemotherapy.

The publication in 1992 of the second EBCTCG overview provides us with reliable data over 10 years of follow-up (Early Breast Cancer Triallists' Collaborative Group, 1992*a*,*b*). It has answered a number of important questions, provided some unexpected results and raised a series of new issues to be addressed by future research, particularly in terms of the potential benefits to be realised by combinations of systemic chemotherapy and hormonal therapies in women of all ages.

The 1992 overview has confirmed the efficacy of chemotherapy for women under the age of 50, and of tamoxifen for women over the age of 50. For women aged less than 50, the use of adjuvant combination chemotherapy reduces the annual odds of recurrence by 37% with a standard deviation of ± 5% (SD5) and the annual odds of death by 27% (SD6). For women over the age of 50, the use of adjuvant tamoxifen reduces the annual odds of recurrence by 30% (SD2) and the annual odds of death by 19% (SD3). Since the vast majority of women who develop recurrent breast cancer ultimately die from the disease, it is anticipated that, after sufficiently long periods of follow-up, the reduction seen in the annual odds of death will match that seen for recurrence. To convert these figures into numbers which are more easily understood, a reduction in the annual odds of death of about 30% equates to approximately 12 extra women alive at 10 years for every 100 women treated with stage II breast cancer, and approximately six extra women alive at 10 years for every 100 women treated with stage I breast cancer. Although these improvements do not appear large, they should be considered in the context of the millions of women treated worldwide each decade for operable breast cancer. Adjuvant treatment of just one million women could well prevent, or substantially delay, an additional 100 000 deaths.

The first overview (Early Breast Cancer Triallists' Collaborative Group, 1990) suggested a limited role for hormonal manipulation in women aged less than 50 with breast cancer. Data from trials of adjuvant tamoxifen suggested a significant reduction in the annual odds of recurrence, but no significant reduction in mortality. Data with respect to ovarian ablation were incomplete.

The 1992 overview has unexpectedly shown that ovarian ablation used as adjuvant therapy in this group of women results in a 30% (SD9) reduction in the annual odds of recurrence and a 28% (SD9) reduction in the annual odds of death. The magnitude of this effect shows no sign of diminishing even after 15 years of follow-up.

More detailed analyses of the tamoxifen trials in women aged less than 50 in the 1992 overview have also provided some surprises with respect to duration of tamoxifen administration. The previous interpretation of these analyses was misleading, since patients treated with both tamoxifen and chemotherapy had a shorter duration of tamoxifen administration (mean 1.6 years) than those treated with tamoxifen alone (mean 2.6 years). Thus, comparisons of tamoxifen duration between trials were likely to have been confounded by the use of chemotherapy. In the 1992 overview, unconfounded analyses of tamoxifen duration in 2216 women treated for a mean of 2.6 years have shown a reduction in the annual odds of recurrence of 27% (SD7) and in the annual odds of death of 17% (SD10), not dissimilar to the results achieved for women over the age of 50. Indeed, further breakdown of these data reveal that, when women aged less than 50 are treated with tamoxifen for 2 years or more, the reduction in the annual odds of recurrence is 43% (SD11), and in the annual odds of death is 27% (SD17) – very similar to those figures described earlier for chemotherapy in this age group. However, it should be noted that because of relatively small numbers in these analyses the confidence intervals are much wider than those in the chemotherapy analyses, implying a degree of statistical instability to the results.

The first overview also suggested a limited role for chemotherapy when given alone to women over the age of 50, for whom a significant reduction in the annual odds of recurrence was demonstrated, but this did not translate into a significant effect on mortality. The 1992 overview, however, clearly shows that chemotherapy does in fact have a significant effect in this age group, reducing the annual odds of recurrence by 22% (SD4), and the annual odds of death by 14% (SD5).

Thus, for women aged less than 50, there are three effective treatments, namely chemotherapy, ovarian ablation and tamoxifen. And for women over the age of 50 there are two effective treatments, namely tamoxifen and chemotherapy. What is not known with any accuracy at the present time is what happens when these treatments are combined and whether the benefits are additive. Data now available from the 1992 overview, shown in Table I, suggest that, for women aged less than 50, tamoxifen may be less effective when given in combination with chemotherapy, in that the additional reductions in the annual odds of recurrence and death from the addition of tamoxifen were only 7% (SD4) and 3% (SD5) respectively when compared with chemotherapy alone

Correspondence: TJ Perren
*P Barrett-Lee, Velindre Hospital, Cardiff; JM Bliss, Institute of Cancer Research, Sutton; J Brown, Brunel University, Uxbridge, Middlesex; AM Brunt, North Staffordshire Royal Infirmary, Stoke; A Cull, Western General Hospital, Edinburgh; S Denton, St Bartholomew's Hospital, London; H Earl, CRC Trials Unit, Birmingham; WD George, Western Infirmary, Glasgow; A Gould, Scottish Cancer Therapy Network, Edinburgh; A Harnett, Beatson Oncology Centre, Glasgow; M Mason, Velindre Hospital, Cardiff; J Mossman, UKCCCR, London; M Richards, Guy's Hospital, London; J Yarnold, Royal Marsden Hospital, Sutton; R Collins (Observer) ICRF Clinical Trials Unit, Oxford.
Received 15 June 1994; revised 15 February 1995; accepted 15 February 1995

**Table I** Estimates from the 1992 overview of reductions in the annual odds of recurrence and death in trials testing combinations of chemotherapy (CT), ovarian suppression (OS) or tamoxifen (Tam)

| *Type of systemic therapy* | *Number of patients* | *Percentage reduction (s.d.) in annual odds of* Recurrence or prior death | Death from any cause |
|---|---|---|---|
| *Age <50 years* | | | |
| (a) CT *vs* CT + OS | 939 | 21 (9) | 19 (11) |
| (b) CT *vs* CT + Tam (mean 1.6 years) | 6362 | 7 (4) | 3 (5) |
| (c) Tam *vs* Tam + CT | 386 | 32 (16) | −6 (23) |
| *Age 50 + years* | | | |
| (d) Tam *vs* Tam + CT | 3932 | 26 (5) | 10 (7) |
| (e) CT *vs* CT + Tam | 8148 | 28 (3) | 20 (4) |

Note that the indicated reductions in the annual odds of recurrence and death are in addition to those reductions already achieved by the therapy in the control arm alone.

(Table Ib). However, these data are based on women receiving tamoxifen for a mean of only 1.6 years. The importance of tamoxifen duration in this age group has been discussed above, and currently it remains unknown whether this apparent inefficacy simply represents an effect of inadequate tamoxifen duration. Since chemotherapy is of proven benefit in women aged less than 50, it might be expected that the addition of chemotherapy to prolonged tamoxifen in this age group would confer additional survival benefits. However, only 386 patients worldwide have been included in such a randomisation (Table Ic). These data do suggest a reduction in the annual odds of recurrence. However, there have been too few events to provide reliable statistical data, particularly concerning survival.

Adjuvant chemotherapy in premenopausal women frequently leads to temporary, or permanent, amenorrhoea. Amenorrhoea is known to occur more frequently in the older pre- or perimenopausal women (Richards *et al.*, 1990). This has led to the suggestion that adjuvant chemotherapy may work via an endocrine mechanism; this is, however, unlikely to be fully accurate since adjuvant chemotherapy is now known to have a clear effect in post-menopausal women. Furthermore, data from 939 patients aged less than 50, who were receiving chemotherapy and also randomised to ovarian suppression, showed a further reduction in the annual risks of recurrence and death of 21% (SD9) and 19% (SD11) respectively, a result that did not quite achieve statistical significance (Table Ia).

There are, however, no data available from randomised trials comparing chemotherapy in combination with ovarian suppression with ovarian suppression alone. Equally, there are also no data on the effects of adjuvant ovarian suppression in patients already receiving tamoxifen. Given that the use of tamoxifen in premenopausal women results in an increase in circulating oestradiol levels, it is feasible that there may be an additional therapeutic benefit from the use of ovarian suppression in such patients. This hypothesis is supported by data in metastatic disease showing responses to tamoxifen in patients who have developed progressive disease following ovarian ablation, and vice versa (Ingle *et al.*, 1986).

In order to resolve these issues, a randomised clinical trial is clearly needed but, as has been demonstrated by the 1992 overview itself, the effects achieved with adjuvant therapies are relatively small and require large patient numbers in order to demonstrate them reliably. The United Kingdom Central Coordinating Committee for Cancer Research (UKCCCR) adjuvant breast cancer (ABC) trial, which was launched in 1993, is a simple and pragmatic trial designed to recruit several thousand patients over a short period of time. It is estimated that in order to demonstrate a 5% difference in survival between arms of the study around 2000 patients will be required for each comparison. The trial has the backing of the Faculty of Clinical Oncology at the Royal College of Radiologists, the Scottish Cancer Trials Breast Group, the West Midlands Breast Group and the Yorkshire Breast Cancer Group. It also has the support of a large number of non-affiliated oncologists entering patients directly through the ABC Trials Office. Some patients have already been entered from overseas and further international collaboration is anticipated. In the UK around 15 000 new cases of breast cancer are diagnosed in women aged less than 70 each year, and with the level of support already demonstrated from the UK and overseas recruitment to the trial should be completed in the next 3–4 years. For those treatment comparisons to which too few patients are randomised to provide definitive evidence on survival, the data generated will make a substantial contribution to future overviews.

Within the trial all patients are treated with prolonged adjuvant tamoxifen. Currently, treatment is recommended for a period of 5 years, but the trial design allows clinicians also to enter their patients into trials of tamoxifen duration should they feel this is appropriate.

The recommendation that all pre- and perimenopausal women should receive tamoxifen is based upon the apparent efficacy of this drug when given in a prolonged fashion to premenopausal women as well as its safety profile. It is also based on pragmatic principles, since many UK clinicians are already using tamoxifen as standard therapy in this group of women.

For pre- or perimenopausal women the clinician decides for each individual patient whether he or she would recommend therapy with either chemotherapy or ovarian suppression in addition to the standard therapy of tamoxifen alone. For premenopausal patients, if uncertainty exists concerning either of these treatments, the clinician is free to offer randomisation to that option. Thus, after discussion and consent, patients may be randomised between chemotherapy and no chemotherapy or between ovarian suppression and no ovarian suppression, or may enter into both randomisations.

For women over the age of 50, the data from the 1992 overview already suggest that tamoxifen and chemotherapy have a largely independent action and that their effect may well be additive (Table Id and e). However, it is as yet uncertain whether the additional toxicity from such combined modality therapy will be worthwhile to individual patients. This is illustrated by the fact that most post-menopausal women with breast cancer in the UK are not currently offered chemotherapy. This question is addressed in the ABC trial through a randomisation between tamoxifen alone and tamoxifen plus chemotherapy.

While the ABC trial is designed to be simple and to keep paperwork to a minimum, it is recognised that the additional discussion required in order to obtain patients' consent to randomisation will significantly increase the workload of participating clinicians. In recognition of this, preliminary funding of £30 000 has been granted by the Medical Research Council to provide participants with a sum of money, on a per capita basis, which can be used to pay for staff to support the running of the trial within individual centres. Examination of the recruitment data shows that the trial is now clearly viable, and by the end of 1994 855 patients had been recruited to the ABC trial, 545 from the UK and 310

from overseas. As a result of successful recruitment a definitive grant is now being sought through the UKCCCR to support the further running of the trial over the next few years.

The success of the ABC trial depends not only on dedicated clinicians, but also on a steady supply of patients who are prepared to give consent to be randomised into the study. To this end the trial has been developed in consultation with organisations representing the interests of patients, including BACUP, Breast Cancer Care and the UK Breast Care Nursing Society. It will also be important to widen the public debate concerning participation of patients in clinical trials. Currently patients are often taken aback when approached concerning a clinical trial; ideally they, should expect to be offered entry into clinical trials. By heightening public awareness and broadening discussion of these items, the ABC trial can make a contribution to achieving this aim.

The advisory group on health technologies has already recognised the importance of well-designed randomised trials to the research and development strategy of the National Health Service. A trial such as the ABC trial has, on the basis of previous research, identified a possible improvement in the treatment of a major health problem, and sets out to test this in a large number of patients. Issues such as quality of life will be incorporated into the trial, as an additional and voluntary module to be conducted in certain centres, as it is clearly important to be able to determine whether the additional toxicities resulting from combined modality treatments are worthwhile in terms of improved outcome for patients. Of particular concern are the long-term toxicities that may result from ovarian ablation in the younger premenopausal women, as well as the shorter term, but nevertheless potentially substantial, toxicities that may result from the addition of chemotherapy to tamoxifen in post-menopausal women. The results of the ABC trial will ultimately be analysed with respect to their impact and cost, allowing calculation of statistics such as number of women-years of life saved, the added costs of treatment and cost savings of a cured patient.

A study of the size of the ABC trial will also facilitate the development of parallel studies to investigate aspects of the biology of breast cancer. Of particular interest is the further investigation and refinement of markers that may predict for chemotherapy or endocrine responsiveness. In premenopausal women there is already a suggestion from a trial that compared adjuvant combination chemotherapy with adjuvant ovarian ablation that the benefits of ovarian ablation were mainly seen in patients with hormone receptor-positive tumours and the benefits of chemotherapy mainly in patients with hormone receptor-negative tumours (Scottish Cancer Trials Breast Group and ICRF Breast Unit, Guys Hospital, London, 1994). Other trials have suggested that CMF-type adjuvant chemotherapy may be most effective in patients with tumours negative for the c-*erb*B-2 oncogene (Allred *et al*., 1992; Gusterson *et al*., 1992), while a trial of dose intensity incorporating adjuvant anthracycline based chemotherapy has suggested that dose-intensive anthracycline chemotherapy has its main effect in patients with tumours positive for the c-*erb*B-2 oncogene (Muss *et al*., 1994). However, all of these studies are relatively small or derived from subgroups of larger studies and require confirmation. Well-conducted biological studies run alongside a trial of the size of the ABC study will be able to contribute significantly to this research. Ultimately the goal must be to identify reliable markers that will allow specific treatments, or combinations of treatments, to be targeted to the individuals most likely to benefit from them. If this can be achieved it will also be possible to spare patients from receiving treatments they do not require, as well as to identify patients not well served by the current generation of adjuvant therapies who will become candidates for experimental therapies. A side-effect of this activity will be the generation of a sizeable well-documented tumour bank which will be available for testing the significance of new markers identified by ongoing fundamental molecular biological and genetic research.

Any clinician currently treating patients with operable breast cancer who is not already entering patients into clinical trials is urged to join the ABC trial. It is only through participation in such studies as this that we will be able further to define optimal treatments for individual patients.

For further information and in order to discover who is coordinating the ABC trial in your region contact Lindsay Johnson by telephone, 0181 643 8901 extension 4188, or fax, 0181 770 7876.

**Acknowledgements**
Dr TJ Perren would like to thank the Imperial Cancer Research Fund and the Yorkshire Cancer Research Campaign for support. The ABC trials office receives funding from the Cancer Research Campaign, and a project grant for per patient payments for the first year of the trial has been awarded by the Medical Research Council.

### References

ALLRED DC, CLARK GM, TANDON AK, MOLINA R, TORMEY DC, OSBORNE CK, GILCHRIST KW, MANSOUR EG, ABELOFF M, EUDEY L AND MCGUIRE WL. (1992). HER-2 neu in node-negative breast cancer: prognostic significance of overexpression influenced by the presence of *in situ* carcinoma. *J. Clin. Oncol.*, **10**, 599-605.

EARLY BREAST CANCER TRIALLISTS' COLLABORATIVE GROUP. (1990). *Treatment of Early Breast Cancer. Vol. 1:* Worldwide Evidence 1985-1990. Oxford University Press: Oxford.

EARLY BREAST CANCER TRIALLISTS' COLLABORATIVE GROUP. (1992*a*). Systemic treatment of early breast cancer by hormonal, cytotoxic, or immune therapy. 133 randomised trials involving 31,000 recurrences and 24,000 deaths among 75,000 women (Part 1). *Lancet*, **339**, 1-15.

EARLY BREAST CANCER TRIALLISTS' COLLABORATIVE GROUP. (1992*b*). Systemic treatment of early breast cancer by hormonal, cytotoxic, or immune therapy. 133 randomised trials involving 31,000 recurrences and 24,000 deaths among 75,000 women (Part 2). *Lancet*, **339**, 71-85.

GUSTERSON BA, GELBER RD, GOLDHIRSCH A, PRICE KN, SäVE-SöDERBORGH J, STYLES J, RUDENSTAM CM, GOLOUH R, REED R, MARTINEZ-TELLO F, TILTMAN A, TORHORST J, GRIGOLATO P, BETTELHEIM R, NEVILLE AM, BüRKI K, CASTIGLIONE M, COLLINS J, LINDTNER J AND SENN H. FOR THE INTERNATIONAL (LUDWIG) BREAST CANCER STUDY GROUP. (1992). Prognostic importance of c-erbB-2 expression in breast cancer. *J. Clin. Oncol.*, **10**, 1049-1056.

INGLE JN, KROOK JE, GREEN SJ, KUBISTA TP, EVERSON LK, AHMAN DL, CHANG MN, BISEL HF, WINDSCHITL HE, TWITO DI AND PFEIFLE DM. (1986). Randomised trial of bilateral oophorectomy versus tamoxifen in premenopausal women with metastatic breast cancer. *J. Clin. Oncol.*, **4**, 178-185.

MUSS HB, THOR AD, BERRY DA, KUTE T, LIU ET, KOERNER F, CIRRINCIONE CT, BUDMAN DR, WOOD WC, BARCOS M AND HENDERSON IC. (1994). c-erB-2 expression and response to adjuvant therapy in women with node-positive early breast cancer. *N. Engl. J. Med.*, **330**, 1260-1266.

RICHARDS MA, O'REILLY SM, HOWELL A, GEORGE WD, FENTIMAN IS, CHAUDARY MA, CROWTHER D AND RUBENS RD. (1990). Adjuvant cyclophosphamide, methotrexate, and fluorouracil in patients with axillary node-positive breast cancer: an update of the Guy's Manchester trial. *J. Clin. Oncol.*, **8**, 2032-2039.

SCOTTISH CANCER TRIALS BREAST GROUP AND ICRF BREAST UNIT, GUYS HOSPITAL, LONDON. (1994). Adjuvant ovarian ablation versus CMF chemotherapy in premenopausal women with pathological stage II breast carcinoma: the Scottish trial. *Lancet*, **341**, 1293-1298.


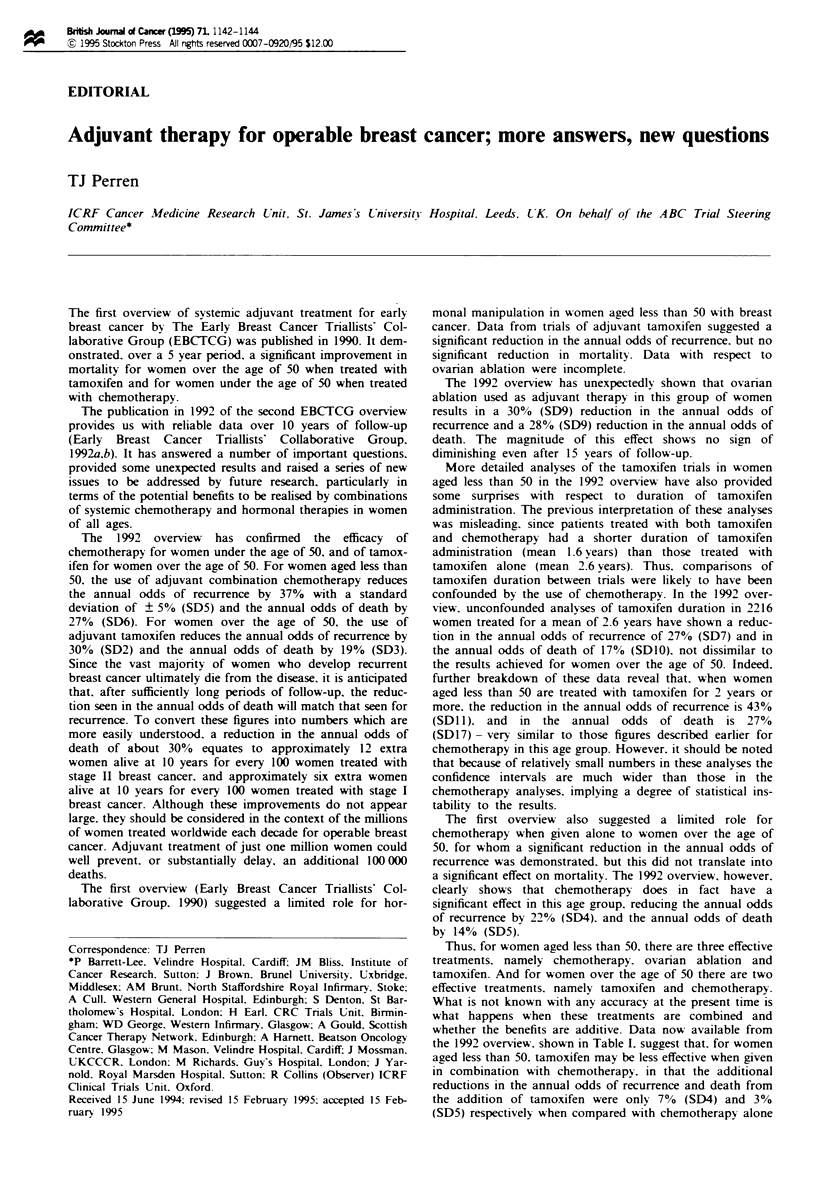

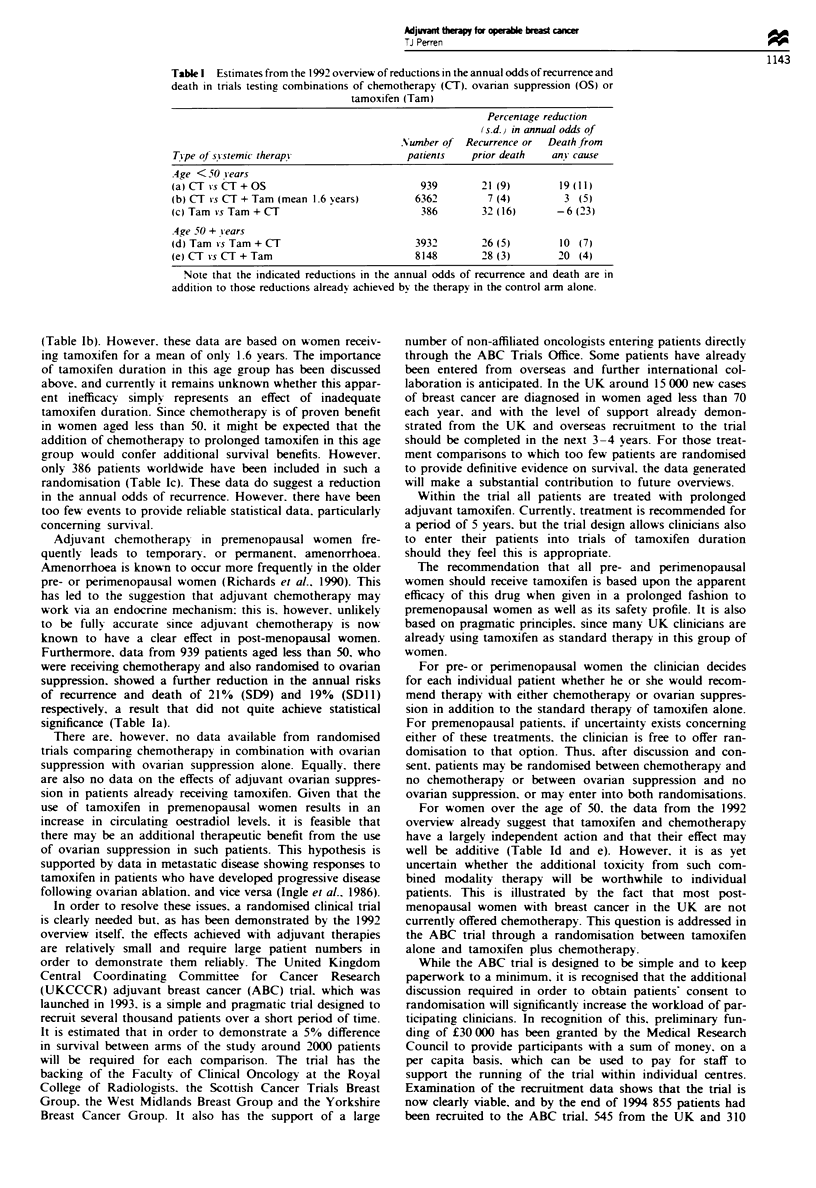

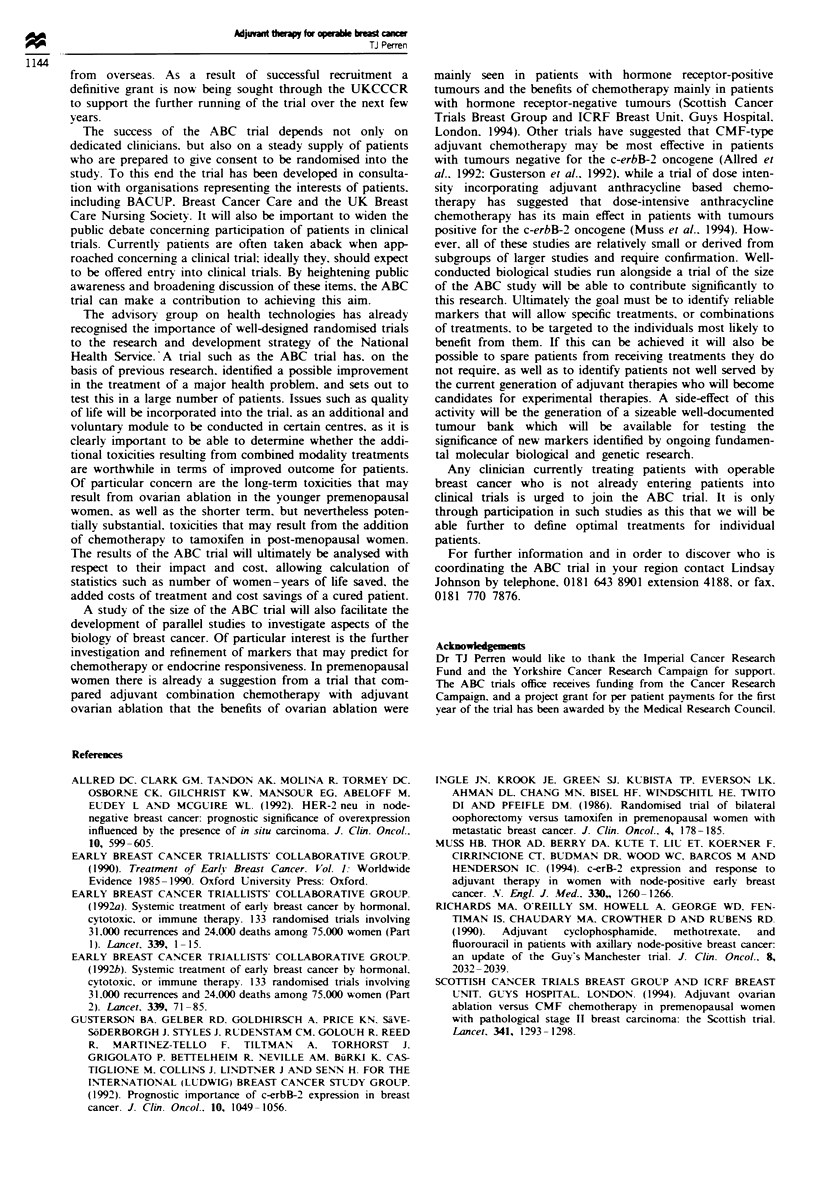


## References

[OCR_00405] Allred D. C., Clark G. M., Tandon A. K., Molina R., Tormey D. C., Osborne C. K., Gilchrist K. W., Mansour E. G., Abeloff M., Eudey L. (1992). HER-2/neu in node-negative breast cancer: prognostic significance of overexpression influenced by the presence of in situ carcinoma.. J Clin Oncol.

[OCR_00438] Ingle J. N., Krook J. E., Green S. J., Kubista T. P., Everson L. K., Ahmann D. L., Chang M. N., Bisel H. F., Windschitl H. E., Twito D. I. (1986). Randomized trial of bilateral oophorectomy versus tamoxifen in premenopausal women with metastatic breast cancer.. J Clin Oncol.

[OCR_00448] Muss H. B., Thor A. D., Berry D. A., Kute T., Liu E. T., Koerner F., Cirrincione C. T., Budman D. R., Wood W. C., Barcos M. (1994). c-erbB-2 expression and response to adjuvant therapy in women with node-positive early breast cancer.. N Engl J Med.

[OCR_00452] Richards M. A., O'Reilly S. M., Howell A., George W. D., Fentiman I. S., Chaudary M. A., Crowther D., Rubens R. D. (1990). Adjuvant cyclophosphamide, methotrexate, and fluorouracil in patients with axillary node-positive breast cancer: an update of the Guy's/Manchester trial.. J Clin Oncol.

